# Automated speech analysis in depressive disorder: Enhancing diagnosis and monitoring

**DOI:** 10.1192/j.eurpsy.2026.10413

**Published:** 2026-07-31

**Authors:** A. Neuberg, M. De Prisco, A. Mas-Musons, C. Valenzuela-Pascual, M. Korniyenko, V. Oliva, G. Fico, J. Raduà, E. Vieta, D. Hidalgo-Mazzei, C. Escolano, G. Anmella

**Affiliations:** 1 Polytechnic University of Catalonia (UPC); 2 Hospital Clínic of Barcelona, Bipolar and Depressive Disorders Unit, IDIBAPS, CIBERSAM, University of Barcelona, Barcelona, Spain; 3 Imaging of Mood- and Anxiety-Related Disorders (IMARD) group, IDIBAPS, Barcelona, Spain., Barcelona, Spain

## Abstract

**Introduction:**

Depression is one of the most prevalent mental disorders and is associated with measurable alterations in speech. Automatic speech analysis offers a promising, non-invasive approach, yet progress is hindered by small, imbalanced datasets and the limited interpretability of many models.

**Objectives:**

This work evaluates acoustic, linguistic, and emotional features for automatic depression recognition, with a focus on balancing strategies, feature selection, and clinically interpretable models.

**Methods:**

We analyzed 185 audio recordings from the DAIC-WOZ dataset, including 55 depressed and 133 non-depressed participants (PHQ-8 ≥ 10). Sixty-one acoustic features (via openSMILE), plus handcrafted linguistic and emotional indicators, were extracted. Six classical classifiers (Decision Tree, Random Forest, SVM, Gradient Boosting, AdaBoost, XGBoost) were trained across 216 configurations combining balancing methods (e.g., SMOTE, SMOTETomek, Random Undersampling) and feature selection approaches. Performance was assessed using accuracy, precision, recall, F1, and ROC-AUC, with emphasis on recall for the depressed class.

**Results:**

Without balancing, models favored the majority class and rarely identified depressed individuals (recall < 0.30). After balancing and feature selection, performance improved substantially. The best model—a Decision Tree with SMOTE oversampling and hybrid feature selection—achieved a weighted F1 score of 0.78 using only eight features. These included prosodic slopes, loudness, response time, valence, and part-of-speech frequencies. Undersampling methods also yielded high recall for depressed participants (up to 0.79).

**Conclusions:**

Interpretable, lightweight models can provide accurate and clinically meaningful predictions of depression from speech. This work establishes a scalable framework for automated mental health assessment and highlights the value of balancing and feature engineering for reliable detection.

**Disclosure of Interest:**

None Declared

